# RFWD2 Knockdown as a Blocker to Reverse the Oncogenic Role of TRIB2 in Lung Adenocarcinoma

**DOI:** 10.3389/fonc.2021.733175

**Published:** 2021-09-27

**Authors:** Ruimin Hao, Jinxia Hu, Yuemei Liu, Dongmin Liang, Yan-Mei Li, Ranran Wang, Shucui Zhang, Pingyu Wang, You-Jie Li, Shuyang Xie

**Affiliations:** ^1^ Department of Biochemistry and Molecular Biology, Binzhou Medical University, Yantai, China; ^2^ Department of Immune Rheumatism, Yantaishan Hospital, Yantai, China; ^3^ Key Laboratory of Cardiovascular Remodeling and Function Research, Qilu Hospital, Shandong University, Jinan, China

**Keywords:** RFWD2, proteasome-mediated degradation, TRIB2, cancer therapy, target gene

## Abstract

RFWD2, an E3 ubiquitin ligase, is overexpressed in numerous human cancers, including leukemia, lung cancer, breast cancer, renal cell carcinoma, and colorectal cancer. The roles of RFWD2 in cancer are related to the targeting of its substrates for ubiquitination and degradation. This study aimed to investigate the role of TRIB2 in relation to the regulation of protein degradation through RFWD2. inBio Discover™ results demonstrated that TRIB2 can perform its functions by interacting with RFWD2 or other factors. TRIB2 can interact with and regulate RFWD2, which further attends the proteasome-mediated degradation of the RFWD2 substrate p-IκB-α. TRIB2 colocalizes with RFWD2-related IκB-α to form a ternary complex and further affects the IκB-α degradation by regulating its phosphorylation. Specific domain analysis showed that TRIB2 may bind to RFWD2 *via* its C-terminus, whereas it binds to IκB *via* its pseudokinase domain. TRIB2 acts as an oncogene and promotes cancer cell proliferation and migration, whereas RFWD2 knockdown reversed the role of TRIB2 in promoting cancer cell growth and colony formation *in vitro* and *in vivo*. In summary, this study reveals that TRIB2 promotes the progression of cancer by affecting the proteasome-mediated degradation of proteins through the interaction with RFWD2.

## Introduction

The ubiquitin (Ub) proteasome system plays important roles in regulating the cell cycle, cell proliferation, cell migration, and apoptosis by affecting the levels and activities of biological function-related proteins (1, 2). Substrate proteins are attached with a small and highly conserved Ub protein consisting of 76 amino acids (3). This attachment reaction is catalyzed by E1 Ub-activating enzyme, E2 Ub-conjugating enzyme, and E3 Ub-ligases (4). Then, the polyubiquitinated proteins are degraded timely by the Ub proteasome system (5), which is essential for maintaining cellular activity and homeostasis. Notably, about 80% of intracellular protein proteolysis depends on Ub, and the dysregulation of Ub proteasome system has been involved in various diseases, including cancer (6). The components of Ub proteasome system are also mutated or abnormally expressed in multiple cancers. E3 Ub ligases can recognize and interact with ubiquity late protein substrates (7), which in several cases lead to the upregulation of oncogenic activities and downregulation of tumor-suppressor activities (8).

To date, about 600 putative E3 ligases have been found in the human genome (9). RFWD2 [also called constitutive photomorphogenic 1 (COP1)] is a RING finger containing protein and operates as an E3 Ub ligase for ubiquitination-mediated degradation by targeting its substrate proteins, thus playing an important role in regulating cell proliferation and apoptosis (10, 11). Based on datasets from Oncomine and Gene Expression Omnibus, RFWD2 is found to be overexpressed in various human cancers, including leukemia, lung cancer, breast cancer, renal cell carcinoma, colorectal cancer, ovarian cancer, and hepatocellular carcinoma (12). RFWD2 knockdown significantly suppresses cell proliferation and induces the apoptosis of human hepatocellular carcinoma cells (13). RFWD2-siRNA treatment can also suppress liver cancer growth and reduce tumor mass in nude mice (13). The overexpression of RFWD2 significantly promotes cell proliferation of human chronic lymphocytic leukemia cells (14). These studies demonstrated that RFWD2 is critically involved in tumorigenesis and may be a novel therapeutic target for cancer therapy.

The tribble (TRIB) pseudokinase protein family consists of three members: TRIB1, TRIB2, and TRIB3 (15). These proteins are predicted to contain three domains: an N-terminal PEST region, pseudokinase domain (containing an unusual N-lobe and canonical C-lobe), and C-terminal RFWD2-binding peptide region, which interacts in cis with a pocket formed adjacent to the unusual C-helix in the TRIB pseudokinase domain (15). Through structure–function analyses, Trib1 and Trib2 may act as adapters to recruit RFWD2-E3 Ub ligase by interacting with C-terminal RFWD2-binding motif (16, 17). In addition, RFWD2 accelerates the development of Trib1- and Trib2-induced acute myeloid leukemia in mouse models, which is abrogated when Rfwd2 or Trib1/2 is deleted or mutated (18, 19). These results suggest that RFWD2 forms a complex with Trib1 or Trib2 to exert its oncogenic roles in AML by promoting the ubiquitination and degradation of its related proteins.

We previously reported that TRIB2 promotes lung cancer development and may play an oncogenic role in lung cancers associated with poor outcomes (20). In this study, inBio Discover™ analysis showed that TRIB2 can perform its functions by interacting with RFWD2. To further study the mechanism of oncogenic TRIB2 and its interaction with RFWD2 in lung cancer, performed immunoprecipitation and immunofluorescence experiments showed that TRIB2 colocalizes with RFWD2-related IκB-α to form a ternary complex and further affected IκB-α degradation by regulating its phosphorylation. TRIB2, as an oncogene, promotes cancer cell proliferation and migration, which can be blocked by knocking down RFWD2.

## Materials and Methods

### Lung Carcinoma Tissues

The experiments were approved by the Medical Ethics Committee of Binzhou Medical University. Severn paired samples of lung adenocarcinoma and adjacent noncancerous lung were obtained from patients who had undergone surgery between October 1, 2018 and July 31, 2019 at Yantaishan Hospital, the Affiliated Hospital of Binzhou Medical University (Yantai, China). Informed consent was obtained from all patients. The tissues were fixed in 10% neutral formalin for immunohistochemistry analysis or extracted with total protein for Western blotting.

### Immunohistochemistry

Immunohistochemistry analysis of paraffin-embedded tissue was performed as follows. The sections were dewaxed with xylene, treated with gradient ethanol for hydration, and repaired with EDTA solution (pH 8.0), followed by blocking with goat serum and incubation with normal IgG. The sections were incubated overnight at 4°C with anti-TRIB2 antibody (1:200 Bioss, Beijing, China), and then incubated in biotin-conjugated second antibody. Proteins were visualized using a pv-9000 two-step detection kit (Zhongshan Jinqiao Biotechnology Co., Ltd., Beijing, China).

### Cell Culture and siRNA Transfection

A549, H1975, and HeLa cells (Shanghai Institute of Cell Biology, Shanghai, China) were cultured in RPMI-1640 medium (Gibco, Grand Island, NY, USA) supplemented with 10% fetal bovine serum (Hyclone, Logan, UT, USA), 100 U/ml penicillin, and 100 μg/ml streptomycin at 37°C in a 5% CO_2_ atmosphere.

Transfection of expression plasmids or short interfering RNAs (siRNAs) into HeLa, A549, and 293T cells was carried out using Lipofectamine™ 3000 (Thermo Fisher Scientific, Waltham, MA, USA; L3000015) according to the manufacturer’s recommendations. All siRNAs were transfected into cells at a final concentration of 50 nM. The sequences of the siRNAs used in this study are shown (**Supplementary Table S1**).

### Western Blotting

Total cell lysates were prepared using RIPA buffer (Beyotime, Shanghai, China). All proteins were separated *via* SDS-PAGE and transferred onto polyvinylidene fluoride membranes (Invitrogen, Carlsbad, CA, USA). The membranes were incubated overnight at 4°C with the following primary antibodies: p-IKB-α, IKB-α, RFWD2, ubiquitin (1:500, Bioworld Technology, Inc., Minneapolis, MN, USA), TRIB2 (1:1,000, Cell Signaling Technology, Danvers, MA, USA), and p-RFWD2 (1:500, Bioss Biotechnology). Next, the membranes were incubated with horseradish peroxidase-labeled secondary antibodies (1:6,000, Beijing Zhong Shan-Golden Bridge Technology Co., Ltd., Beijing, China), and the signals were detected with an Automatic Image Analysis System (Tanon 5200 Multi, Shanghai, China) following electrochemiluminescence immune reactions.

### Plasmids

The DNA sequences of TRIB2-full and its truncated mutants were amplified using pcDNA-TRIB2 as a template. The primer sequences used are as shown in **Supplementary Table S2**. These DNA fragments were inserted into the p3×flag-CMV-9-10 vector plasmid. To construct the GFP-TRIB2-expressing plasmid, the cDNA for TRIB2 was amplified from pcDNA-TRIB2 using the same primers with Flag-TRIB2-full *via* PCR and ligated into the pEGFP-C3 plasmid containing an N-terminal GFP tag.

### Immunoprecipitation

Cells were seeded into 10-cm plates and transfected with Flag-tagged expression plasmid vectors using Lipofectamine 2000 (Invitrogen) for 48 h. The total lysate was extracted with NP-40 buffer (50 mM Tris-Cl at pH 7.4, 150 mM NaCl, 1 mM EDTA, 1% NP-40, 0.5% SDS, protease inhibitor mixture) and incubated with 50 µl of Anti-Flag M2 Affinity Gel (Sigma-Aldrich, St. Louis, MO, USA) at 4°C for 2 h. Following centrifugation, the samples were washed with PBS and heated with 40 µl 1× loading buffer, and the supernatant was subjected to SDS-PAGE followed by immunoblotting.

To analyze the interactions among endogenous proteins, 500 µg of cell extracts were incubated with primary antibodies or control IgG overnight at 4°C, and then with protein G/A beads (Invitrogen) for 2 h at 4°C. The beads were washed with lysis buffer, mixed with protein loading buffer, and detected by SDS-PAGE.

### Immunofluorescence Detection

Cells growing on glass coverslips were fixed with 4% paraformaldehyde, permeabilized with 0.1% NP-40, and incubated with rabbit antihuman IKB-α/RFWD2 (1:100; Bioworld Technology) overnight at 4°C. The cells were then incubated with Alexa Fluor 488 donkey antirabbit IgG (H+L) or Alexa Fluor 594 donkey antimouse IgG (H+L) (Molecular Probes, Eugene, OR, USA) at 37°C for 1 h. Immunofluorescence was observed using a microscope (DM6000B, Leica, Wetzlar, Germany).

### 3-(4,5-Dimethythiazol-2-yl)-2,5-Diphenyl tetrazolium Bromide Assay

Cells transfected with siRNAs (GenePharma, Shanghai, China; siRNA sequences were shown in **Supplementary Table S1**) or pcDNA-TRIB2 plasmids were cultured in 96-well plates for 48 h as described previously (20). Next, 10 µl 3-(4,5-dimethythiazol-2-yl)-2,5-diphenyl tetrazolium bromide (MTT; 5 mg/ml, Sigma-Aldrich) was added to the medium in each well. The medium was removed 4 h later, and 100 µl dimethyl sulfoxide (Sigma-Aldrich) was added, and the OD value (570 nm) was detected with a microplate reader (Multiskan FC, Thermo Fisher Scientific). Each experiment was performed in triplicate and repeated at least three times.

### Analysis of Apoptotic Cells

Harvested cells were treated with Annexin V-FITC/PI (KeyGEN Biotech. Co., Ltd., Nanjing, China) according to the manufacturer’s instructions and counted by flow cytometry (Beckman Coulter, Brea, CA, USA). Each experiment was performed in triplicate and repeated at last three times.

### Colony-Formation Assay

Cells transfected with indicated siRNAs or plasmids were maintained in culture media for 14 days, followed by staining with crystal violet. Colonies containing more than 20 cells were counted. Each experiment was performed in triplicate and repeated at last three times.

### Migration Assays

Cells (10^4^ cells per well in 100 µl fetal bovine serum-free 1640 medium) were seeded into the upper chamber of a Corning Costar Transwell chamber (Sigma-Aldrich), and 600 µl of RPMI-1640 medium containing 20% calf serum was added to the lower chamber. After 16 h, cells in the upper chamber were removed using cotton swabs, and migrated cells in the lower chamber were fixed with 4% paraformaldehyde and incubated with 1% crystal violet (Sigma) for 15 min. After washing with ddH_2_O, five fields per Transwell were examined under a microscope (DM6000B, Leica). The assay was repeated three times for each group.

Migration assays were also performed using an xCELLigence DP instrument following the manufacturer’s instructions (ACEA Biosciences Inc., San Diego, CA, USA); 1.5 × 10^5^ cells in 100 µl of medium were seeded into the upper compartment of a CIM plate in triplicate per group, after which 160 µl medium containing 20% fetal bovine serum was added to the lower compartment. The cell index, representing the number of migrated cells, was calculated *via* RECA (real-time cell analysis) software 1.2.1 (ACEA Biosciences).

### A549 Lung Adenocarcinoma Cell Xenografts

Animals were grouped by simple randomization of using random number table. A549 cells stably expressing TRIB2 or treated with TRIB2-siRNA or RFWD2-siRNA were harvested, and 2 × 10^6^ cells were injected subcutaneously into the backs of female BALB/C-nude mice aged 6–8 weeks (HFK Bio-Technology, Beijing, China) as previously described (20). The second siRNA was injected into the xenografts on day 14 after the initial treatment. Tumor volumes were measured daily using calipers. The animals were sacrificed by intraperitoneal injection of a barbiturate at 30 days after injection. All animal experiments were approved by the Committee on the Ethics of Animal Experiments of Binzhou Medical University.

### Statistical Analysis

SPSS 22.0 software (IBM Corp., Armonk, NY, USA) was used to analyze statistical significance. Data were tested for normal distribution using a normality test. Data are expressed as mean ± SD. The Student’s *t*-test was used to compare two averages. ANOVA was used for mean comparison of multiple groups; depending on whether the assumption of homogeneity of variance was satisfied or not, the LSD test or the Games-Howell test was used to compare means of different samples. Abnormally distributed data were expressed as median (interquartile range); the Mann-Whitney *U* test and Kruskal-Wallis H test were used to compare two groups or multiple groups, respectively. Statistical significance was set at *P* < 0.05.

## Results

### TRIB2 Promotes Cancer Cell Proliferation and Migration

**Figure 1:**
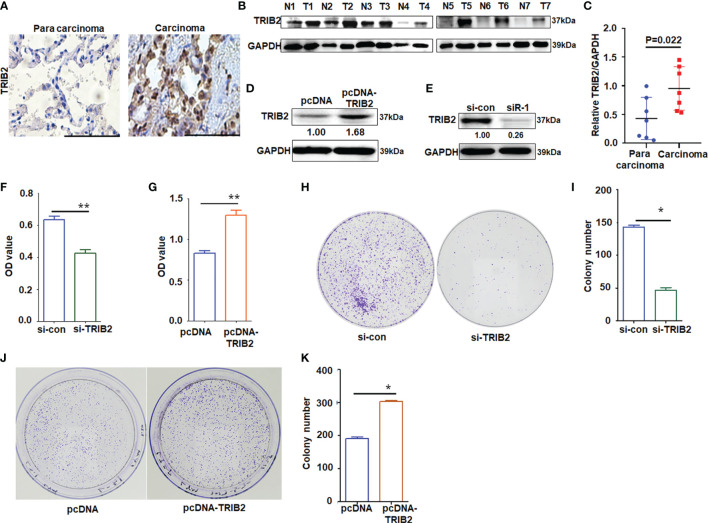
Effects of TRIB2 on cancer cell proliferation. **(A)** Immunohistochemistry analysis of TRIB2 expression in one paired sample of lung anticarcinoma versus adjacent normal lung tissue; bar = 100 μm. **(B)** Total proteins from seven paired samples of lung adenocarcinoma (*T*) versus adjacent normal tissues (*N*) were extracted for Western blotting analysis with antibodies against TRIB2. **(C)** The levels of TRIB2 in seven lung adenocarcinoma and paired normal lung tissues of **(B)** was quantified with Image J (NIH, Bethesda, MD, USA) software and normalized to GAPDH, data were expressed as median (interquartile range). **(D, E)** TRIB2 expression in A549 cells treated with pcDNA-TRIB2 or TRIB2-siRNA and controls was assessed by Western blotting. GAPDH was used as the loading control. **(F)** TRIB2-siRNA inhibits A549 cell viability by MTT assay following siRNA transfection for 48 h Data represent the mean ± SD; for triplicate experiments. **p < 0.01; LSD test. **(G)** Overexpression of TRIB2 increases viability of A549 cells by MTT assay. Data represent the mean ± SD; for triplicate experiments. **p < 0.01; LSD test. **(H, I)** Knocking down TRIB2 inhibits colony formation of A549 cells. Data represent the mean ± SD; for triplicate experiments. *p < 0.05; LSD test. **(J, K)** Overexpression of TRIB2 increased colony formation of A549 cells. Data represent the mean ± SD; for triplicate experiments. *p < 0.05; LSD test.

*TRIB2* acts as an oncogene in various tumors. Our previous study demonstrated that the overexpression of TRIB2 is related to the poor survival of patients with lung cancer, and miR-206 and miR-140 induce lung cancer cell death and suppress cell proliferation by regulating oncogenic TRIB2 promoter activity through p-Smad3 (20). To further determine the mechanism of TRIB2 in the progression and development of lung adenocarcinoma (LUAD), we detected the expression of TRIB2 in lung cancer and adjacent normal lung tissues by immunohistochemistry analysis. TRIB2 was overexpressed in lung cancer tissue compared with the adjacent normal lung tissue (**Figure 1A**). Next, we collected seven lung cancer and adjacent normal lung-tissue samples for Western blotting to detect the expression of TRIB2. We observed that TRIB2 was overexpressed in LUAD (**Figures 1B, C**). In a previous study, we transfected A549 cells with three pairs of siRNA-targeting TRIB2 and confirmed their effectiveness for knocking down TRIB2 (20). Next, we transiently transfected pcDNA-3.1-TRIB2 or siRNA into A549 cells to determine the role of TRIB2 in cancer after its overexpression or downregualtion (**Figures 1D, E**). The results of an MTT assay, which was performed to clarify the role of TRIB2 in cancer progression, showed that TRIB2 knockdown inhibited the proliferation of A549 cells (**Figure 1F**), whereas TRIB2 overexpression promoted the proliferation of A549 cells (**Figure 1G**). Plate colony formation indicated that cell clones, which were smaller and less abundant following TRIB2 knockdown in A549 cells (*p* < 0.01; **Figures 1H, I**), became larger and more abundant after TRIB2 overexpression (*p* < 0.01; **Figures 1J, K**).

Flow cytometry detection was conducted to investigate whether TRIB2-regulated cell proliferation is related to apoptosis. The results obtained demonstrated that the apoptotic rate of A549 cells significantly increased following TRIB2 knockdown but decreased following TRIB2 overexpression (**Supplementary Figures S1A, B**). Tumor cells often exhibit strong migration capabilities. Cell migration experiments indicated that TRIB2 knockdown significantly inhibited cell migration compared with that in the control group (**Supplementary Figures S1C, D**; *p* < 0.05). Following TRIB2 overexpression, the number of migrated cells increased significantly (**Supplementary Figures S1E, F**; *p* < 0.05), suggesting that TRIB2 promoted the migration of lung cancer cells. This finding was also supported by cell migration experiments using HeLa cells (**Supplementary Figure S2**).

### TRIB2 Interacts With RFWD2

**Figure 2:**
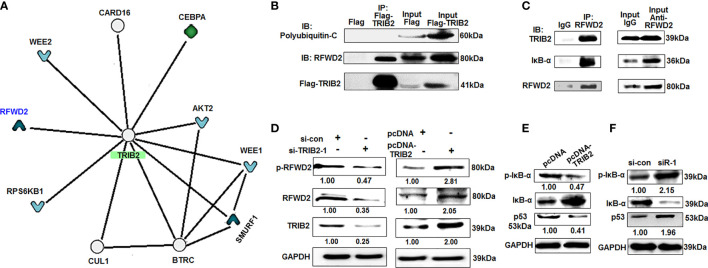
TRIB2 interacts with RFWD2. **(A)** The results of inBio Discover™ showed that TRIB2 may interact with RFWD2, CEBPA, WEE2, etc. **(B)** 293T cells were transiently transfected with Flag-TRIB2 or Flag vector for 48 h, and then the total lysate was coimmunoprecipitated using M2 affinity gel. **(C)** Whole-cell lysate of 293T cells was immunoprecipited with RFWD2 antibody followed by immunoblotting with antibodies against indicated proteins. **(D)** TRIB2 positively regulates the expression of RFWD2 and si-TRIB2 inhibits p-RFWD2 and RFWD2 levels, as shown by Western blotting analysis. Expression levels of RFWD2 and TRIB2 quantified *via* ImageJ software and normalized to GAPDH. **(E, F)** Expression levels of IκB-α, p-IκB-α, and p53 in A549 cells treated with pcDNA-TRIB2 or TRIB2-siRNA and controls assessed by Western blotting. GAPDH was used as the loading control.

To investigate the mechanism of TRIB2 in promoting cancer cell proliferation, the interactions between TRIB2 and other proteins were analyzed with inBio Discover™ online (https://inbio-discover.com/#login). With inBio Discover™, we can explore the interactions between proteins, and gain valuable biological functions. The inBio Discover™ results demonstrated that TRIB2 can perform its functions by interacting with RFWD2, C/EBPα, WEE2, etc. (**Figure 2A**). TRIB2 is also specifically regulated by Wnt signaling in liver cancer cells, which is associated-Ub E3 ligase-TrCP, RFWD2, and Smurf1 reducing TCF4/β-catenin expression (21). To further verify whether the roles of TRIB2 are related to RFWD2 or other Ub E3 ligases, we extracted the total protein from 293T cells transiently expressing Flag-TRIB2 and performed coimmunoprecipitation experiments using an M2 affinity chromatography column binding anti-Flag antibodies. Our results confirmed the interaction between TRIB2 and RFWD2 but did not support the interaction between TRIB2 and polyubiquitin-C (**Figure 2B**). Coprecipitation experiments with the total lysates of 293T cells showed that anti-RFWD2 antibodies precipitated RFWD2, together with TRIB2 (**Figure 2C**). These results indicate that TRIB2 can interact with RFWD2, which may be related to TRIB2-regulating cancer cell proliferation.

### TRIB2 With RFWD2 Attends Proteasome-Mediated Degradation of Proteins

RFWD2 is an E3 Ub ligase that plays a vital role in the regulation of cell proliferation and apoptosis (12). The abovementioned results showed that TRIB2 can interact with RFWD2, which indicates that the function of TRIB2 may affect the proteasome-mediated degradation of proteins through the interaction between TRIB2 and RFWD2. To investigate this hypothesis, we first detected the effect of TRIB2 on RFWD2 levels after the overexpression or knockdown of TRIB2 by Western blot. The levels of RFWD2 were significantly reduced following TRIB2 knockdown for 48 h in A549 cells but increased following TRIB2 overexpression (**Figure 2D**). These results indicate that the role of TRIB2 is related to its interaction with and effects on RFWD2. The roles of RFWD2 in cancer is related to targeting its substrates for ubiquitination and degradation, such as p-IκB-α (22), p53, Jun, STAT3, β-catenin, p27, and C/EBPα (12). Next, we detected the levels of RFWD2-related ubiquitination substrate p-IκB-α and p53 after the overexpression or knockdown of TRIB2 by Western blot. Our results showed that TRIB2 overexpression inhibited the p-IκB-α and p53 levels, whereas TRIB2 knockdown resulted in their increase (**Figures 2E, F**). The abovementioned results indicate that TRIB2 can affect p-IκB-α and p53 levels by regulating RFWD2.

**Figure 3:**
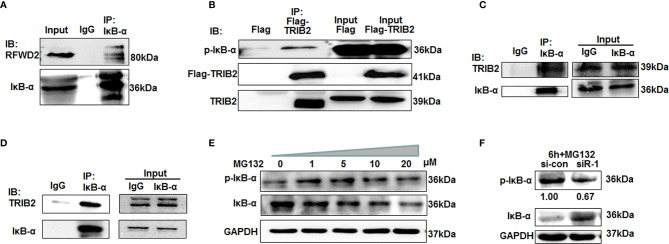
TRIB2 with RFWD2 attend proteasome-mediated p-IκB-α degradation. **(A)** Coimmunoprecipitation with IKB-α antibodies showing interaction between RFWD2 and IκB-α in 293T cells. **(B)** Coimmunoprecipitation experiments were carried out using total lysates of 293T cells overexpressing Flag and Flag-TRIB2. M2 affinity gel precipitated TRIB2 together with p-IκB-α. **(C)** Coimmunoprecipitation using 293T lysate and anti-IκB-α antibodies. TRIB2 and IκB-α precipitated together. **(D)** Expressed and purified eukaryotic TRIB2 and IκB-α proteins used for coimmunoprecipitation experiments to further study the interaction between IκB-α and TRIB2. **(E)** p-IκB-α and IκB-α expression detected by Western blotting in A549 cells following treatment with various concentrations of MG132. **(F)** A549 cells were treated with 20 μM MG132 for 6 h following TRIB2 knockdown with siRNA and the total lysate was subjected to Western blotting to detect p-IκB-α and IκB-α expression levels.

To further investigate the above hypothesis, we selected IκB-α as a research object for coprecipitation experiments with the total lysates of 293T cells. The results showed that anti-RFWD2 antibodies precipitated RFWD2, also together with IκB-α. Further coimmunoprecipitation experiments indicated that IκB-α also interacted with RFWD2 (**Figures 2C**, **3A**). To determine whether TRIB2 interacts directly with IκB-α, we conducted coimmunoprecipitation with cells overexpressing Flag-tagged TRIB2. The results demonstrated that anti-Flag M2 affinity gel precipitated TRIB2 with p-IκB-α (**Figure 3B**). We next confirmed the endogenous interaction between TRIB2 and p-IκB-α in 293T cells (**Figure 3C**). We further used eukaryotic expressed and purified TRIB2 and IκB-α proteins in coimmunoprecipitation experiments and observed that IκB-α antibodies may precipitate IκB-α and TRIB2 (**Figure 3D**). These results indicate that TRIB2, IκB-α, and RFWD2 form a ternary complex with RFWD2 though direct physical interactions, which may be related to the effect of TRIB2 on the proteasome-mediated degradation of proteins with RFWD2.

Prior studies also supported that TRIB2 regulates the degradation of certain proteins *via* the Ub-proteasome pathway (23, 24). To further test whether TRIB2 can regulate the degradation of IκB *via* the Ub–proteasome pathway, we treated A549 cells with various concentrations of MG132, an inhibitor of the proteasome degradation pathway. Western blotting indicated that p-IκB-α expression increased in an MG132 concentration-dependent manner (**Figure 3E**). The findings confirmed that IκB-α was phosphorylated and entered the ubiquitination degradation pathway. However, IκB-α degradation was blocked when A549 cells were treated with 20 μM MG132 following TRIB2 knockdown with siRNA, suggesting that TRIB2 affected IκB-α degradation by regulating its phosphorylation (**Figure 3F**).

### TRIB2 Colocalizes With RFWD2-Related IκB-α

**Figure 4:**
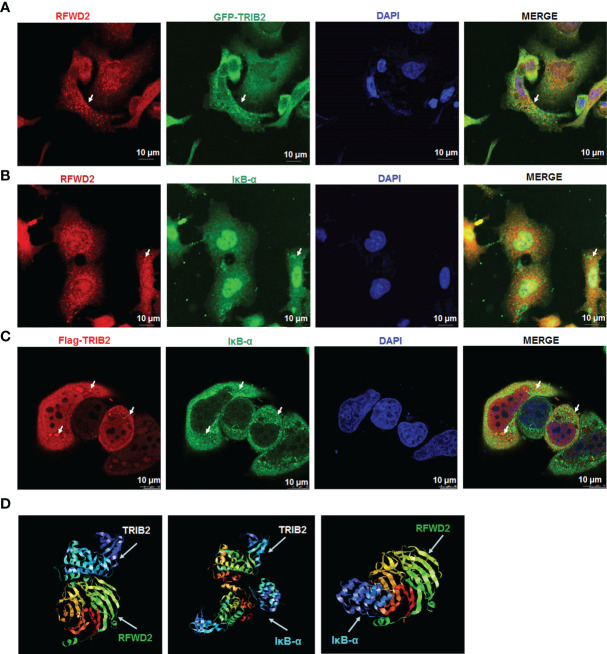
Colocation analysis between TRIB2, RFWD2, and IκB-α. **(A)** Colocalization of RFWD2 (red) and GFP-TRIB2 (green) in A549 cells, observed *via* confocal microscopy. Immunostaining was performed with primary RFWD2 antibody after A549 cells were treated with GFP-TRIB2. Scale bar = 10 µm. **(B)** Immunostaining of A549 cells with antibodies against RFWD2 (red) and IκB-α (green). Scale bar = 10 µm. **(C)** A549 cells transfected with Flag-TRIB2 and subjected to immunostaining with antibodies against IκB-α (green) and TRIB2 (red). Scale bar = 10 µm. **(D)** Interactions between TRIB2, IκB-α, and RFWD2 analyzed *via* the PRODIGY Tool.

To test whether TRIB2 affects the proteasome-mediated degradation of protein IκB-α through a ternary complex TRIB2-RFWD2-IκB-α, we performed immunofluorescence staining to evaluate the interactions and colocalization among TRIB2, RFWD2, and IκB-α. Laser confocal microscopy revealed partial colocalization of exogenously expressed GFP-TRIB2 with FRWD2 in A549 cells (**Figure 4A**) and colocalization of endogenously expressed IκB-α with RFWD2 (**Figure 4B**). Following the transfection of A549 cells with Flag-TRIB2, immunofluorescence analysis revealed the colocalization of Flag-TRIB2 with IκB-α (**Figure 4C**). Altogether, these results support that TRIB2, IκB-α, and RFWD2 form a ternary complex. We also used a network tool (PRODIGY, https://nestor.science.uu.nl/prodigy/) to model the three-dimensional structures of TRIB2 and IκB-α and analyzed their potential for interaction with RFWD2. The PRODIGY results indicated that these three proteins directly interact (**Figure 4D**; **Supplementary Table S3**).

### Mapping the Interaction of TRIB2, RFWD2, and IκB-α

**Figure 5:**
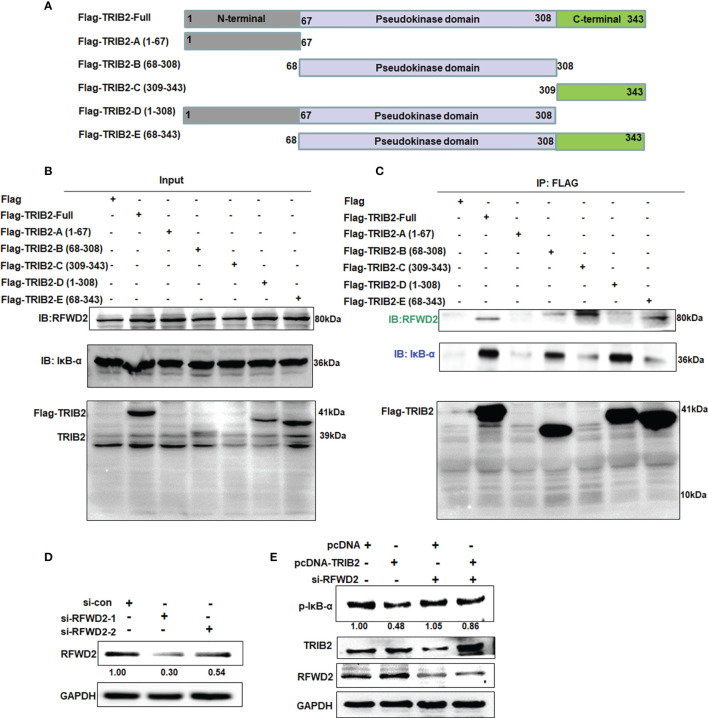
Mapping interaction of TRIB2 with IκB-α or RFWD2. **(A)** Diagram showing constructs of Flag-TRIB2 and its deletion mutants. **(B, C)** Coimmunoprecipitation analysis of the domains of TRIB2 involved in the interaction with RFWD2 and IκB-α in 293T cells treated with Flag-TRIB2 deletion mutants. **(D)** Expression levels of RFWD2 were decreased in RFWD2-siRNA-treated A549 according to Western blotting. RFWD2 expression was normalized to GAPDH. **(E)** RFWD2 knockdown attenuated the role of TRIB2 in regulating the expression of p-IκB-α, as shown by Western blotting. Relative intensities of p-IκB-α were normalized to that of GAPDH.

TRIB2 contains an N-terminal PEST region, pseudokinase domain, and C-terminal region (25). To identify the specific domain associated with the interaction of TRIB2 with RFWD2 and IκB-α, we constructed Flag-tagged TRIB2-domain deletion mutants (**Figure 5A**) and transiently transfected them into 293T cells. After 48 h, coimmunoprecipitation analysis of the total protein extract showed that RFWD2 precipitated together with Flag-TRIB2-Full, Flag-TRIB2-C, and Flag-TRIB2-E, indicating that TRIB2 interacted with RFWD2 mainly *via* its carboxyl terminus (**Figures 5B, C**). Flag-TRIB2-Full, Flag-TRIB2-B, and Flag-TRIB2-D coprecipitated with IκB-α, indicating that the pseudokinase domain is mainly required for the interaction between TRIB2 and IκB-α. Because of the affection of the binding of RFWD2 and the structure of mutant Flag-TRIB2-E, a relatively weak band was found in Flag-TRIB2-E. The abovementioned results indicate that TRIB2 may bind to RFWD2 *via* its C-terminus, whereas it binds to IκB *via* its pseudokinase domain. Therefore, the combination of TRIB2 with RFWD2 causes no effect on its interaction with IκB-α.

These above results indicate that TRIB2 can regulate the proteasome-mediated degradation of RFWD2. To further understand the biological role of RFWD2 in TRIB2 affecting the proteasome-mediated degradation of RFWD2-related protein, we screened siRNAs that effectively knock down RFWD2 (**Figure 5D**). siRNAs specific for RFWD2 were cotransfected with the control vector or TRIB2 expression plasmids into A549 cells, and the expression of related proteins was detected. The results showed that the levels of RFWD2-related protein p-IκB-α increased following the RFWD2 knockdown compared with the control treatment (**Figure 5E**). Our results indicate that the function of TRIB2 in regulating proteasome-mediated degradation requires the participation of RFWD2.

### TRIB2 Promotes Cell Proliferation and Migration in a RFWD2-Dependent Manner

**Figure 6:**
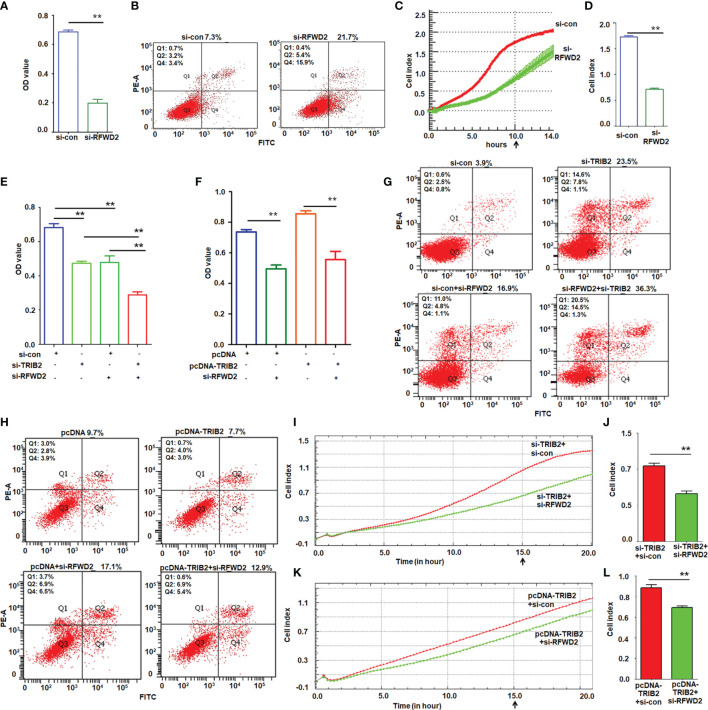
TRIB2 promotion of cell growth and migration depends on RFWD2. **(A)** MTT assay showing that RFWD2 downregulation inhibited A549 cell proliferation. Data were expressed as the mean ± SD from triplicate experiments. **p < 0.01; Student’s *t*-test. **(B)** Apoptotic rate of A549 cells transfected with RFWD2-siRNA for 48 h measured *via* flow cytometry from triplicate experiments. **(C, D)** RFWD2 knockdown inhibited cell migration. A549 cells treated with siRNAs were analyzed *via* a real-time migration assay on an xCELLigence RTCS. Data are expressed as the mean ± SD of triplicate experiments. **p < 0.01; Student’s *t*-test. **(E)** Viability of A549 cells transfected with the indicated siRNAs analyzed *via* MTT assay. Data are expressed as the mean ± SD of triplicate experiments. **p < 0.01; ANOVA. **(F)** Cell viability of A549 cells following treatment with the indicated plasmids or siRNAs, as analyzed by MTT assay. Data are expressed as the mean ± SD of triplicate experiments. **p < 0.01; ANOVA. **(G)** TRIB2 and RFWD2 downregulation promotes apoptosis, as shown by flow cytometry assay performed in triplicate. **(H)** Cell apoptosis induced by RFWD2 knockdown is rescued by TRIB2 overexpression as evaluated in triplicate. **(I, J)** Real-time migration assay on an xCELLigence RTCS showed that simultaneous knockdown of RFWD2 and TRIB2 inhibited migration more strongly than TRIB2 knockdown alone. Cell index represents the mean ± SD of triplicate experiments. **p < 0.01; Student’s *t*-test. **(K, L)** Real-time migration assay on an xCELLigence RTCS showed that TRIB2 overexpression recused migration inhibition caused by RFWD2 knockdown. Cell index represents the mean ± SD of triplicate experiments. **p < 0.01; Student’s *t*-test.

The abovementioned results showed that TRIB2 promoted cell proliferation and migration, and that the function of TRIB2 in regulating gene expression requires the participation of RFWD2. We next evaluated whether blocking the RFWD2 activity suppresses the oncogenic role of TRIB2. Using siRNA to knockdown RFWD2, we investigated the effect of RFWD2 on the proliferation and migration of A549 cells. MTT assay showed that proliferation was significantly inhibited in siRNA-treated cells compared with that in the control oligo treatment group (*p* < 0.01; **Figure 6A**). Fluorescence-activated cell sorting assay indicated that the apoptosis rate was elevated in siRNA-treated cells (**Figure 6B**). Real-time cell analysis of migration further showed that RFWD2 knockdown inhibited cell migration (**Figures 6C, D**; *p* < 0.01). The results in H1975 cells also supported that siRNA-RFWD2 inhibited cell proliferation and migration, and increased lung cancer cell apoptosis (**Supplementary Figure S3**). These results indicate that RFWD2 knockdown suppresses A549 cell proliferation.

Next, we analyzed the effect of RFWD2 on TRIB2-induced cell proliferation and migration. The MTT assay suggested that either TRIB2-siRNA or RFWD2-siRNA suppressed the proliferation of A549 cells; this suppressive effect was evident when both proteins were downregulated simultaneously (**Figure 6E**). Although TRIB2 overexpression promoted cell proliferation, knockdown of RFWD2 partially weakened the oncogenic role of *TRIB2* in A549 cells (**Figure 6F**). The apoptosis assay demonstrated that although the knockdown of either RFWD2 or TRIB2 individually increased the apoptosis rate, knocking them both down significantly increased the apoptotic rate of A549 cells (**Figure 6G**). Moreover, RFWD2 downregulation partially attenuated the role of TRIB2 (**Figure 6H**). The RTCA migration assay demonstrated that the simultaneous knockdown of TRIB2 and RFWD2 suppressed cell migration more substantially than the knockdown of TRIB2 alone (**Figures 6I, J**), and that knockdown of RFWD2 reduced the role of TRIB2 in cell migration (**Figure 6K, L**). The results in H1975 cells further supported that siRNA-RFWD2 can reverse the role of TRIB2 in cell proliferation, migration, and apoptosis (**Supplementary Figure S3**).

A plate colony-formation assay showed that RFWD2 knockdown inhibited the colony-forming ability of A549 cells (**Supplementary Figures S4A, B**); TRIB2 had the same effect. However, when both TRIB2 and RFWD2 were knocked down simultaneously, the suppressive effect on colony formation became much more remarkable (**Supplementary Figures S4C, D**). Moreover, RFWD2 knockdown reversed the role of TRIB2 in promoting colony formation (**Supplementary Figures S4E, F**). These results indicate that the oncogenic role of TRIB2 is inseparable from RFWD2.

### RFWD2 Level Is High in Cancer and RFWD2-siRNA Blocks TRIB2 Functions *In Vivo*

**Figure 7:**
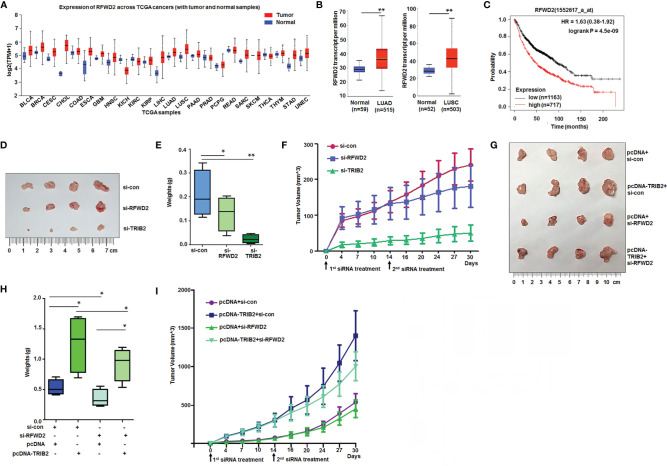
High levels of RFWD2 in cancer and RFWD2 inhibition blocking functions of TRIB2 *in vivo.* **(A)** RFWD2 is obviously higher in the tissues of various kinds of tumors. **(B)** RFWD2 is overexpressed in lung adenocarcinoma and lung squamous cell carcinoma tissues. **(C)** Kaplan-Meier plot analysis of the relationship between RFWD2 levels and clinical outcome. **(D)** Suppressive role of TRIB2-siRNA and RFWD2-siRNA on A549 cell xenografts in BALB/C-nude mice. **(E)** The xenografts of each group (*n* = 4) were weighed, and data were expressed as median (interquartile range). *p < 0.05, **p < 0.01. **(F)** Volumes of xenografts from each group measured over time. **(G)** Effect of RFWD2 downregulation in xenografts of A549 cells stably overexpressing TRIB2. **(H)** Xenografts from each group (*n* = 4) were weighed, and data were expressed as median (interquartile range). **p < 0.05. **(I)** Volumes of xenografts from each group measured over time.

The abovementioned results demonstrated that RFWD2 knockdown suppressed cancer cell proliferation. To further analyze its roles in tumorigenesis, we investigated the RFWD2 expression in cancer based on datasets from The Cancer Genome Atlas online (http://ualcan.path.uab.edu/). The analyzed results demonstrated that RFWD2 is remarkably upregulated in the tissues of numerous kinds of tumors, including bladder urothelial carcinoma (BLCA), breast invasive carcinoma (BRCA), cervical squamous cell carcinoma (CESC), cholangiocarcinoma (CHOL), esophageal carcinoma (ESCA), glioblastoma multiforme (GBM), stomach adenocarcinoma (STAD), etc., compared with control tissues (**Figure 7A**). RFWD2 is also overexpressed in LUAD and lung squamous cell carcinoma (LUSC) tissues compared with normal tissues (**Figure 7B**). Kaplan-Meier plot analysis further (http://kmplot.com/analysis/) demonstrated that high RFWD2 levels from patients with lung cancer were related to extremely poor clinical outcomes (**Figure 7C**). These results indicate that RFWD2 may play an important role in promoting lung cancer cell proliferation.

Furthermore, siRNA- and control-treated cells were injected subcutaneously into the backs of BALB/C-nude mice to produce xenografts to investigate the effects of TRIB2 and RFWD2 on the tumorigenesis of lung cancer cells *in vivo*. On day 14, similar doses of siRNA and control were injected into the xenografts. After 4 weeks, the volume of transplanted tumors in the siRNA-TRIB2 and siRNA-RFWD2 groups was considerably smaller than that in the control group (**Figures 7D, E**). The tumor growth curve further demonstrated that the downregulation of TRIB2 and RFWD2 inhibited tumor formation (**Figure 7F**).

To further verify whether the oncogenic role of *TRIB2* is inseparable from RFWD2 *in vivo*, we knocked down RFWD2 in A549 cell xenografts stably overexpressing TRIB2. The weight and growth curve of the xenografts showed that TRIB2 overexpression promoted tumor formation, whereas RFWD2 knockdown inhibited tumor formation and reduced tumorigenesis in xenografts stably expressing TRIB2 (**Figures 7G–I**). These results suggest that RFWD2 is important for the oncogenic roles of TRIB2 *in vitro and in vivo*.

## Discussion

**Figure 8:**
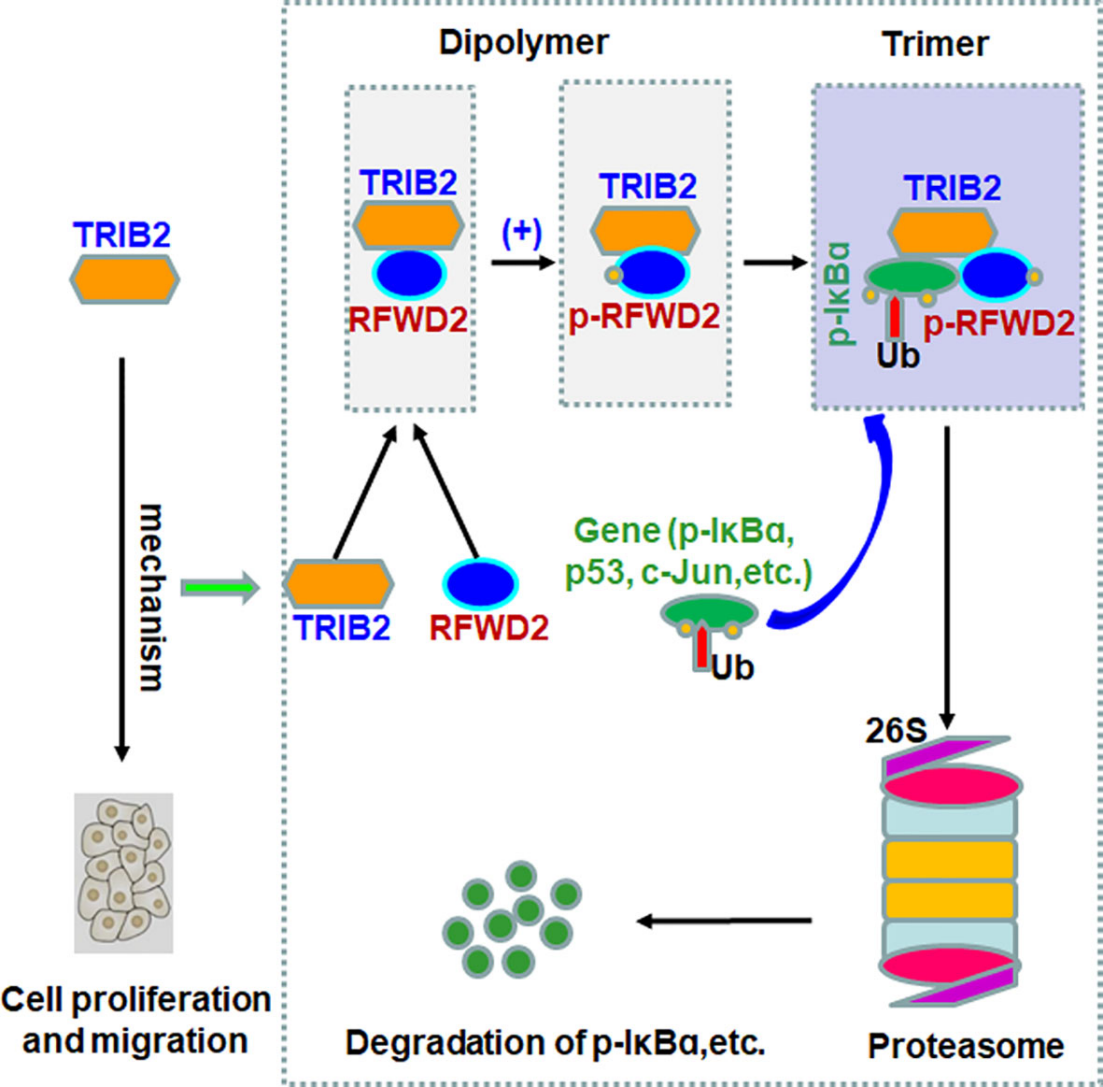
Proposed model of the mechanism by which oncogenic roles of TRIB2 with RFWD2 in regulating proteasome-mediated degradation of proteins.

TIRB2 is an oncogene involved in the development of several tumors, such as melanoma, colorectal cancer, acute myeloid leukemia, and liver cancer (25–28). In addition, the overexpression of TRIB2 in tumor tissues induces drug resistance by promoting phospho-AKT (at Ser473) *via* its COP1 domain. The highest TRIB2 protein expression from patients with cancer is related with an extremely poor clinical outcomes (29). Previously, we observed that TRIB2 promoted the proliferation and migration of lung cancer cells *in vitro* and *in vivo* (30, 31). The current study further verified whether the oncogenic role of TRIB2 depends on RFWD2 in regulating lung cancer cell proliferation. Our results showed that TRIB2 can promote cancer cell proliferation and migration, interact with RFWD2, and regulate RFWD2-related gene expression in lung cancer cells. Furthermore, our results indicated that TRIB2, RFWD2, and IκB-α form a ternary complex, which may be related to the effect of TRIB2 on the proteasome-mediated degradation of proteins with RFWD2. Therefore, we propose that TRIB2 may promote lung cancer cell proliferation *via* the following mechanisms (**Figure 8**): At first, TRIB2 binds RFWD2-E3 Ub ligase based on C-terminal RFWD2-binding motif to form a dipolymer, which increases phosphorylated RFWD2 levels. Next, RFWD2-E3 ubiquitin ligase further recruits and confers its substrate (p-IκB-α or others) specificity for Ub ligation. Then, TRIB2, RFWD2, and substrate p-IκB-α form a ternary complex, following which TRIB2 may regulate and drive the RFWD2-mediated degradation of the substrate *via* the ubiquitination pathway.

RFWD2, a ring-finger-type ubiquitin E3 ligase, was originally identified as a central regulator of photomorphogenesis in *Arabidopsis thaliana* (32). RFWD2 is highly conserved among different vertebrates and has been identified in mice and humans (33). The human RFWD2 gene is located on chromosome 1q24.1 (34). RFWD2 protein structure possesses three highly conserved domains, namely, a RING-finger region (N-terminal), a coiled-coil domain, and C-terminal domain with seven WD40 repeats, which are related to the modulation of protein–protein interactions (34, 35). Human RFWD2 shuttles between the cytoplasm and nucleus, which depends on distinct signals (36). Evidence shows that mammalian RFWD2 is expressed in the heart, lung, bladder, brain, pancreas, liver, spleen, kidney, aorta, skeletal muscle, ovary, prostate, and other tissues of humans and mice (34, 37). RFWD2 is also overexpressed in various kinds of human cancers (12), such as leukemia, lung cancer, breast cancer, lymphoma, glioma, melanoma, colorectal cancer, etc. We observed that RFWD2 levels were higher in lung cancer tissues compared with those in normal controls, and high levels of RFWD2 from patients with lung cancer showed a poor survival outcome. SiRNA-RFWD2 inhibited the proliferation of A549 and Hela cells and promoted the apoptosis and migration of cells. Our results showed that RFWD2 may play an important role in promoting lung cancer cell proliferation.

RFWD2, directly or indirectly, promotes protein degradation (38), and the substrates for human RFWD2 include the following: p-IκB-α (22); p53 (11, 39); C/EBPα (18); 14-3-3σ (40); β-catenin (12); activator protein-1 and c-Jun (10, 41); forkhead box protein O1 (42); p27Kip1 (12); transducer of regulated CREB activity 2 (43); and metastasis-associated protein (44). The roles of RFWD2 in cancer may be related to its promotion of the degradation of these proteins. We observed that siRNA-RFWD2 inhibited the proliferation of A549 and Hela cells and promoted the apoptosis and migration of cells, indicating that RFWD2 play important roles in tumorigenesis. We also revealed that RFWD2 interacts with p-IκB-α, and TRIB2 can affect p-IκB-α levels through regulating RFWD2. TRIB2, RFWD2, and IκB-α form a ternary complex with RFWD2 through direct physical interactions. Our results also demonstrated that the function of TRIB2 in regulating proteasome-mediated degradation of p-IκB-α requires the participation of RFWD2.

TRIB2 participates in the regulation of Wnt/β-catenin signaling (21), AP4/p21 signaling, and E2F1-C/EBPα feedback loop (26, 45). In most cases, the function of TRIB2 is related to the regulation of protein degradation. TRIB2 interacts with E3 Ub ligases (Smurf1, RFWD2, or β-TrCP), which further regulates the ubiquitination and degradation of specific protein signals (23). Moreover, the relationship between TRIB2 and the Ub proteasome system is complex, and may involve competition or cooperation with E3 ubiquitin ligase in binding TRIB2. Phosphorylated TRIB2 is also a target protein of Smurf1 and β-TrCP (46, 47), and it participates in the ubiquitination of TRIB2 and subsequently cause the degradation of TRIB2 to proteasome. In this study, inBio Discover™ results demonstrated that TRIB2 can perform its functions by interacting with RFWD2 or other factors. Next, we discovered that TRIB2 can regulate RFWD2 by interaction. The role of RFWD2 in cancer is related to the targeting of its substrates, such as p-IκB-α, for ubiquitination and degradation (22). Although IκBα is a substrate of E3 Ub ligases and TRIB1 knockdown inhibits the phosphorylation and degradation of IκBα (48), the relationship and regulation between TRIB2 and IκBα are unclear. All these results inspired us to study the effect of TRIB2 on IκB-α. We observed that TRIB2 overexpression increased the RFWD2 levels in cancer cells, indicating that the regulating action of TRIB2 may be related to its interaction with and effect on RFWD2. To further explore its role in TRIB2-promoted tumorigenesis in cancer, we used siRNA to knock down RFWD2. Our results demonstrated that RFWD2 knockdown inhibited the proliferation of A549 and Hela cells and promoted the apoptosis and migration of cells. We recorded that RFWD2 knockdown attenuated TRIB2-promoted colony formation. These data further suggest that RFWD2 assists TRIB2 in promoting cancer progression, and cooperates with TRIB2 to participate in the proteasome-mediated degradation of the RFWD2 substrate p-IκB-α.

Although the present study elucidated a potential mechanism through which TRIB2 interacts with E3 Ub ligases (RFWD2) and further regulates the ubiquitination and degradation of p-IκB-α *via* the TRIB2-IκB-α-RFWD2 ternary complex, several issues associated with this process remain unclear. Prior studies have reported that TrCP and RFWD2 may bind the C-terminus of TRIB2. TRIB2, RFWD2, and TrCP are involved in the degradation of β-catenin. Therefore, the involvement of TrCP in TRIB2 promoted IκB-α ubiquitination and degradation, and the competitive or synergistic nature of the role played by RFWD2 and TrCP in regulating IκBα degradation may require further study. Moreover, IκB-α is an important factor in NF-kB signaling pathway and the RFWD2-TRIB2 complex can regulate IκBα degradation, the effect of RFWD2 or TRIB2 on NF-kB signaling pathway also needs further study.

## Data Availability Statement

The original contributions presented in the study are included in the article/**Supplementary Material**. Further inquiries can be directed to the corresponding authors.

## Ethics Statement

The studies involving human participants were reviewed and approved by the Medical Ethics of committee of Binzhou Medical University. The patients/participants provided their written informed consent to participate in this study. The animal study was reviewed and approved by the Ethics of Animal Experiments of Binzhou Medical University.

## Author Contributions

SX and Y-JL conceived the study and participated in the study design. SX, PW, RH, JH, YL, DL, Y-ML, RW, and SZ designed and performed most assays, analyzed data, and wrote the manuscript. SX, PW, RH, and Y-JL analyzed RNA expression. Y-JL, Y-ML, and SZ provided technical support. All authors contributed to the article and approved the submitted version.

## Funding

The present study was supported by the National Natural Science Foundation of China (No. 81772281, 31371321, 81502536), Shandong Science and Technology Committee (No. ZR2019MH022, ZR2020KH015), Education Department of Shandong Province (2019KJK014), Yantai Science and Technology Committee (2018XSCC051), and Shandong Province Taishan Scholar Project (ts201712067).

## Conflict of Interest

The authors declare that the research was conducted in the absence of any commercial or financial relationships that could be construed as a potential conflict of interest.

## Publisher’s Note

All claims expressed in this article are solely those of the authors and do not necessarily represent those of their affiliated organizations, or those of the publisher, the editors and the reviewers. Any product that may be evaluated in this article, or claim that may be made by its manufacturer, is not guaranteed or endorsed by the publisher.
