# Supplementary material for: RFWD2 Knockdown as a Blocker to Reverse the Oncogenic Role of TRIB2 in Lung Adenocarcinoma
Source: Front Oncol. 2021 Sep 27;11:733175. doi: 10.3389/fonc.2021.733175 (PMC8503262; doi:10.3389/fonc.2021.733175)
Supplement: Supplementary file 1 [file DataSheet_1.pdf]

Supplemental Tables and Figures

Supplemental Tables 1-3.

Table S1. The sequence of siRNAs used in this study

siRNA	5′ to 3′
TRIB2-siRNA1	UAGCGAGAUAUGGGAGAUC
TRIB2-siRNA2	CCCAGGAUUUCGAAGAGUUG
TRIB2-siRNA3	CUUGUCGCAUUGCGUUUCUUG
RFWD2-siRNA1	GGACCACUCAGUGAGUAGCA
RFWD2-siRNA2	GCUGUGGUCUACCAAUCUA
siRNA control	CCUACGCCACCAAUUUCGU

Table S2. The primer sequences for plasmid construction in this study

Fragments	Primer sequences
Flag-TRIB2-Full	Sense: CCCAAGCTTatgaacatacacaggtc<br>Antisense: CGCGGATCCtcagttaaagaaagggtc
Flag-TRIB2-A	Sense: CCCAAGCTTatgaacatacacaggtc<br>Antisense: CGCGGATCCccaacaataagtatttcc
Flag-TRIB2-B	Sense: CCCAAGCTTaacctctggagggagacc<br>Antisense: CGCGGATCCaaaaccaaggatggtcc
Flag-TRIB2-C	Sense: CCCAAGCTTctacagattttagcgtc<br>Antisense: CGCGGATCCtcagttaaagaaagggtc
Flag-TRIB2-D	Sense: CCCAAGCTTatgaacatacacaggtc<br>Antisense: CGCGGATCCaaaaccaaggatggtcc
Flag-TRIB2-E	Sense: CCCAAGCTTaacctctggagggagacc<br>Antisense: CGCGGATCCtcagttaaagaaagggtc
GFP-TRIB2	Sense: CCCAAGCTTatgaacatacacaggtc<br>Antisense: CGCGGATCCtcagttaaagaaagggtc

Table S3. The prediction of binding affinity in biological complexes

**Protein-protein complex**	**ΔG (kcal mol^{-1})**	**K_d (M) at 25.0 ℃**	**K_d (M) at 37.0 ℃**
**Trib2--RFWD2**	**-9.8**	**6.7×10^{-08}**	**1.3×10^{-07}**
**Trib2--IKB-α**	**-9.8**	**6.5×10^{-08}**	**1.2×10^{-07}**
**RFWD2--IKB-α**	**-12.4**	**8.4×10^{-10}**	**1.9×10^{-09}**

Supplemental Figures 1-3.

Figure S1

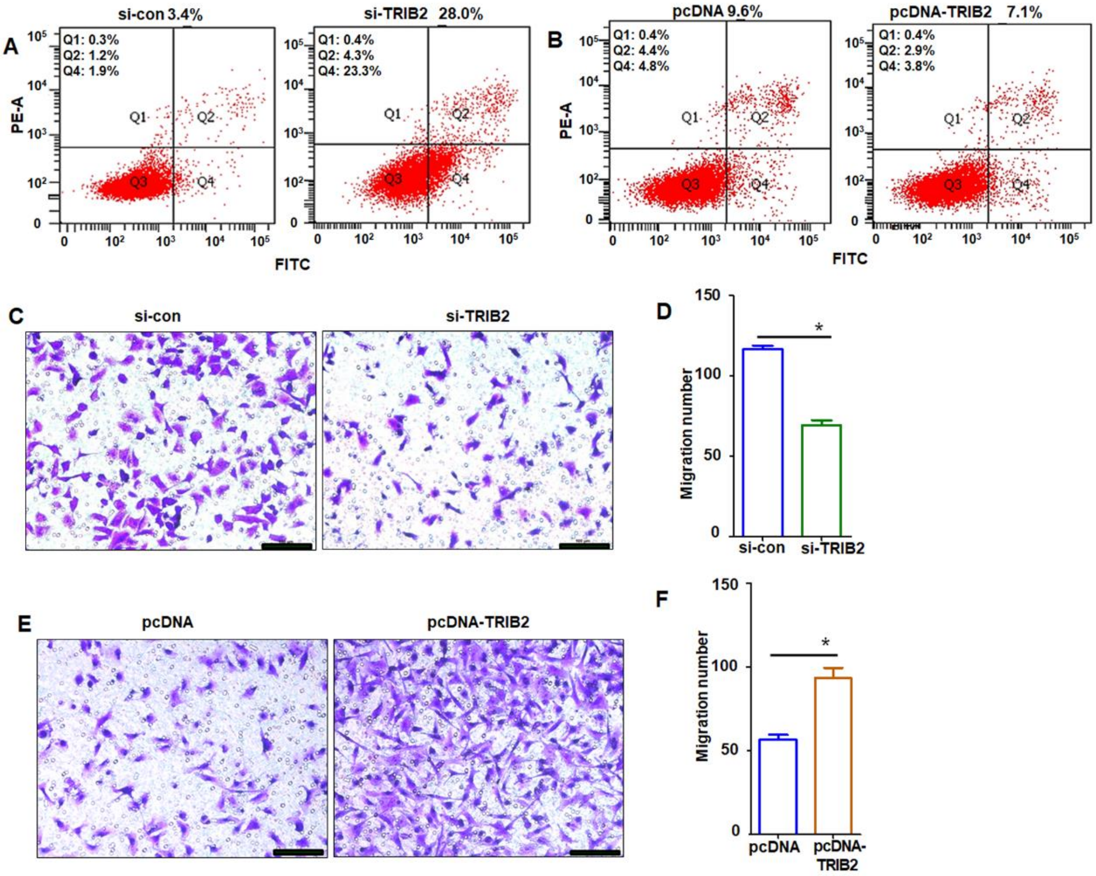

Figure S1 Effects of TRIB2 on cell apoptosis and migration. (A,B) The effect of downregulation or upregulation of TRIB2 on A549 cell apoptosis by FACS analysis for triplicate experiments. **(C,D)** TRIB2 downregulation inhibits cell migration of A549 cells transfected with siRNA for 48h. bar=100μm. Data represent the mean±SD; for triplicate experiments. *P<0.05; LSD test. **(E,F)** Overexpression of TRIB2 promotes cell migration. bar=100μm. Data represent the mean±SD; for triplicate experiments. *P<0.05; LSD test.

Figure S2

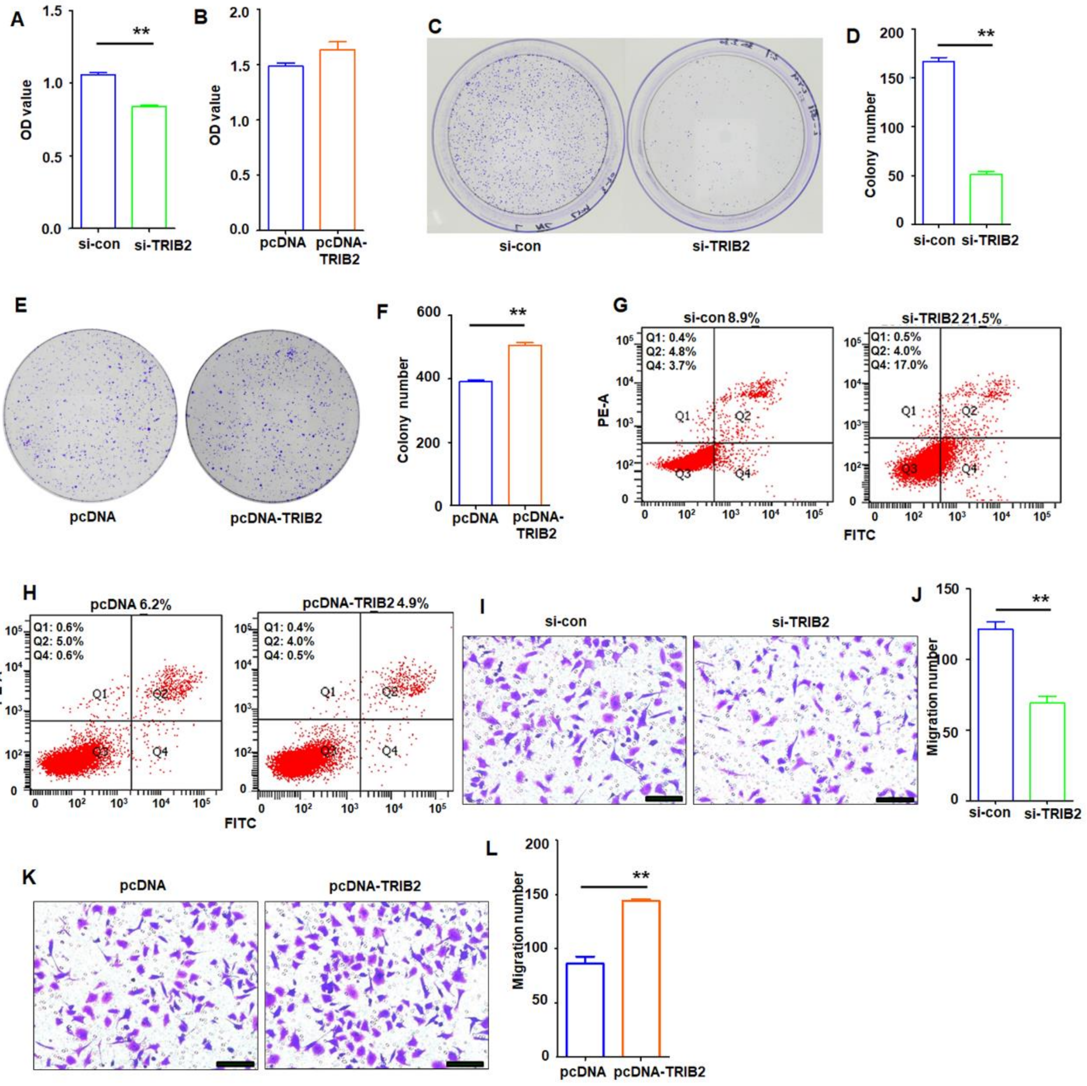

Figure S2. TRIB2 promotes HeLa cell proliferation and migration.

(A) MTT assay showing that TRIB2-siRNA inhibits the viability of HeLa cells transfected with siRNAs for 48 h. Data are expressed as the mean ± SD of triplicate experiments. **p < 0.01; Student's *t*-test. **(B)** MTT assay showing that TRIB2 overexpression increases the viability of HeLa cells transfected with pcDNA-TRIB2 for 48 h. Data are expressed as the mean ± SD of triplicate experiments. **p < 0.01; Student's *t*-test. **(C, D)** TRIB2 downregulation inhibits colony formation of HeLa

cells. Data are expressed as the mean ± SD of triplicate experiments. **p < 0.01; Student's *t*-test. **(E, F)** TRIB2 overexpression increases the colony formation of HeLa cells. Data are expressed as the mean ± SD of triplicate experiments. **p < 0.01; Student's *t*-test. **(G, H)** Fluorescence-activated cell sorting analysis conducted in triplicate indicating the effect of downregulation or upregulation of TRIB2 on A549 cell apoptosis. **(I, J)** TRIB2 downregulation inhibits cell migration of HeLa cells transfected with siRNA for 48 h. bar =100 μm. Data are expressed as the mean ± SD of triplicate experiments. **p < 0.01; Student's *t*-test. **(K, L)** Overexpression of TRIB2 promotes HeLa cell migration. Bar = 100 μm. Data are expressed as the mean ± SD of triplicate experiments. **p < 0.01; Student's *t*-test.

Figure S3

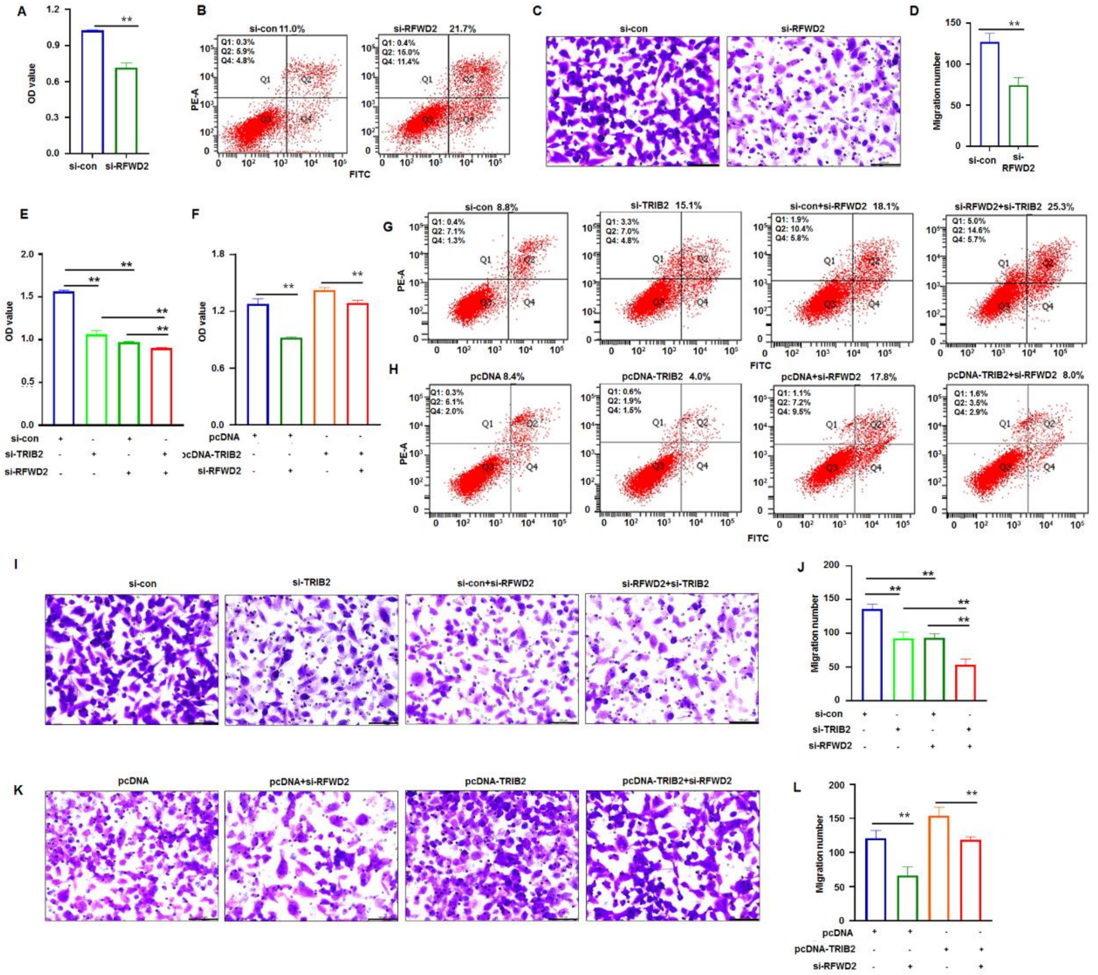

Figure S3 siRNA-RFWD2 blocked the oncogenic role of TRIB2 in H1975 cells.

(A) MTT assay. RFWD2 downregulation supressed H1975 cell proliferation. Data were expressed as the mean ± SD from triplicate experiments. **p < 0.01; Student's t-test. (B) Apoptotic rate of H1975 cells transfected with RFWD2-siRNA for 48 h measured via flow cytometry from triplicate experiments. (C, D) RFWD2 knockdown inhibited H1975 cell migration. Data are expressed as the mean ± SD of triplicate experiments. **p < 0.01; Student's t-test. (E) MTT assay. TRIB2-siRNA or RFWD2-siRNA inhibited H1975 cell proliferation. Data are expressed as the mean ± SD of triplicate experiments. **p < 0.01; ANOVA. (F) MTT assay. Knocking RFWD2 down weakened the role of *TRIB2* in H1975 cells. Data are expressed as the mean ±

SD of triplicate experiments. **p < 0.01; ANOVA. (G) siRNA-TRIB2 and siRNA-RFWD2 induce H1975 cell apoptosis performed in triplicate. (H) Knocking RFWD2 down weakened the role of *TRIB2* in apoptotic rate of H1975 cells as evaluated in triplicate. (I, J) Knockdown of RFWD2 and TRIB2 inhibited migration more strongly than only TRIB2 knockdown. Data are expressed as the mean ± SD of triplicate experiments. **p < 0.01; ANOVA. (K, L) TRIB2 overexpression recused migration inhibition caused by RFWD2 knockdown. Data are expressed as the mean ± SD of triplicate experiments. **p < 0.01; Student's t-test.

Figure S4

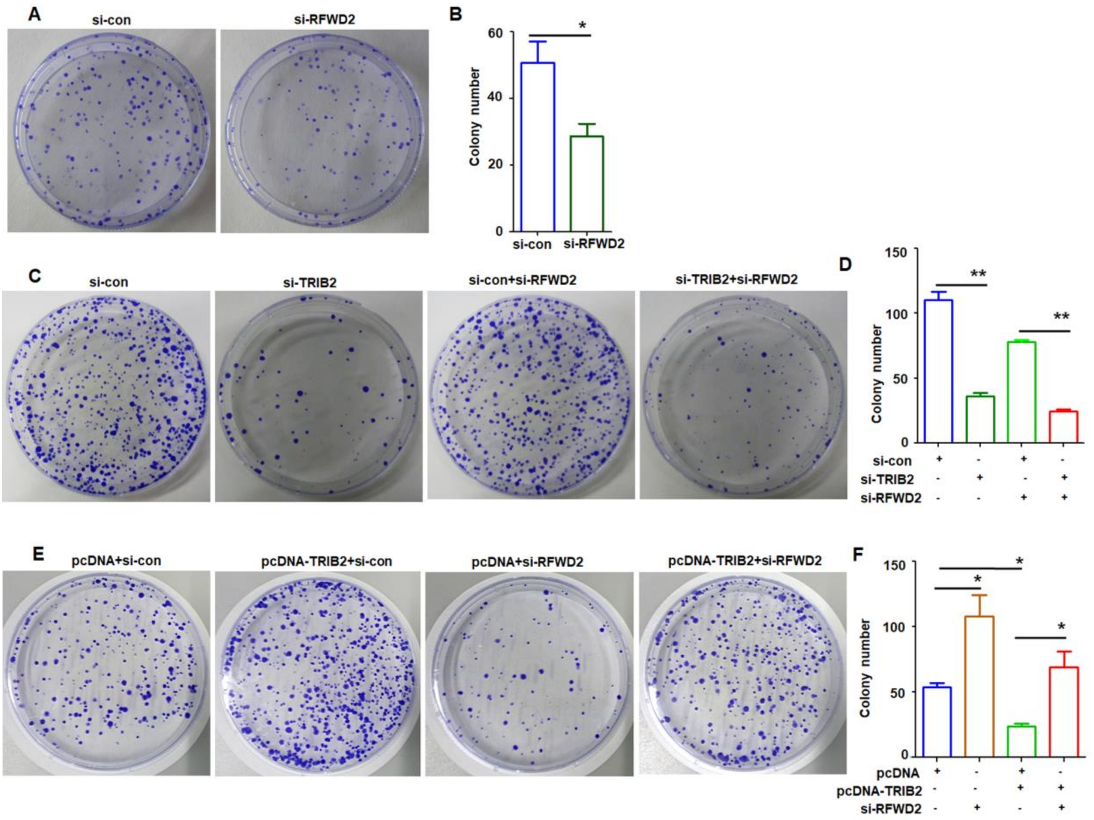

Figure S4. Effects of RFWD2 and TRIB2 on cell colony formation.

(A, B) RFWD2 knockdown inhibits colony formation in A549 cells. Data are expressed as the mean ± SD of triplicate experiments. *p < 0.05; Student's *t*-test. **(C, D)** Colony-formation assays were carried out with A549 cells transfected with the indicated siRNAs. Data are expressed as the mean ± SD of triplicate experiments. **p < 0.01; ANOVA. **(E, F)** Effect of RFWD2 downregulation attenuates TRIB2-promoting colony formation. Data are expressed as the mean ± SD of triplicate experiments. **p < 0.01; ANOVA.
